# Challenges and Prospects for Helper-Dependent Adenoviral Vector-Mediated Gene Therapy 

**DOI:** 10.3390/biomedicines2020132

**Published:** 2014-04-02

**Authors:** Pasquale Piccolo, Nicola Brunetti-Pierri

**Affiliations:** 1 Telethon Institute of Genetics and Medicine, Naples 80131, Italy; E-Mail: piccolo@tigem.it; 2 Department of Translational Medicine, Federico II University of Naples, Naples 80131, Italy

**Keywords:** adenovirus, helper-dependent adenoviral vectors, gutless vectors, liver directed gene therapy

## Abstract

Helper-dependent adenoviral (HDAd) vectors that are devoid of all viral coding sequences are promising non-integrating vectors for gene therapy because they efficiently transduce a variety of cell types *in vivo*, have a large cloning capacity, and drive long-term transgene expression without chronic toxicity. The main obstacle preventing clinical applications of HDAd vectors is the host innate inflammatory response against the vector capsid proteins that occurs shortly after intravascular vector administration and result in acute toxicity, the severity of which is dose dependent. Intense efforts have been focused on elucidating adenoviral vector–host interactions and the factors involved in the acute toxicity. This review focuses on the recent acquisition of data on such interactions and on strategies investigated to improve the therapeutic index of HDAd vectors.

## 1. Introduction

Adenoviruses (Ad) are the most commonly used vectors in human clinical trials and approximately 75% of these trials are directed to cancer treatment [1]. Replication-defective Ad vector expressing p53 is now widely used in China for therapy of cancer. Ad vectors are also used to treat cardiovascular diseases, such as artery diseases, angina, and ischemia. In addition, they have been employed as a genetic vaccine to treat or prevent infections. 

Ad have a non-enveloped icosahedral capsid containing a linear double-stranded DNA genome of ~30–40 kb. Up to now, at least 55 different human Ad (species A–G) have been reported and they are generally associated in immunocompetent patients with subclinical or self-limiting diseases of the airways, eyes, and gastrointestinal tract. Ad vectors most commonly used for clinical trials and experimental gene therapy applications, including helper-dependent adenoviral (HDAd) vectors, are derived from serotypes 2 (Ad2) and 5 (Ad5) of subgroup C (reviewed in [2]). The Ad genome is flanked by inverted terminal repeats (ITRs) which are the only sequences required in *cis* for viral DNA replication. A *cis*-acting packaging signal, required for encapsidation of the genome, is located near the left ITR. The Ad genome can be roughly divided into two sets of genes: the early region genes (E1A, E1B, E2, E3, and E4) that are expressed before DNA replication and the late region genes (L1 to L5) that are expressed to high levels after DNA replication. First generation Ad (FGAd) vectors typically have foreign DNA inserted in place of early region 1 (E1). E1-deleted vectors are replication deficient and are propagated in E1 complementing cells such as 293 [3]. Transgene expression by FGAd vectors is only transient *in vivo* because of acute and chronic toxicity secondary to low levels of viral gene expression from the vector backbone [4]. In contrast, HDAd vectors are devoid of all viral coding sequences and can drive long-term transgene expression in the absence of chronic toxicity [5]. These vectors can be generated through the Cre/loxP system [6] and are produced in large amounts through the method developed by Palmer and Ng [7,8,9]. 

HDAd vectors hold great potential for a large number of therapeutic applications including both inherited and acquired diseases [10,11,12,13], and genetic vaccination [14]. Moreover, they are promising vectors for cancer immunotherapy, as discussed in the paper by Suzuki in this special issue [15]. Previous reviews [5,16,17] have focused on the wide range of preclinical applications of HDAd vectors whereas the scope of this paper is to present the general features of HDAd vectors, the recent acquisitions on Ad vector-host interactions, and the strategies to overcome the problem of vector-induced acute toxicity. 

## 2. Intravascular Delivery of Ad Vectors

Intravascular delivery of Ad vectors is performed to target the liver or in the context of cancer gene therapy to achieve larger vector distribution to the tumor site(s). Moreover, the liver is a very attractive target for gene therapy because it is the affected organ in several genetic and acquired diseases and it can be used as a factory organ for systemic delivery through the circulation of vector-encoded therapeutic proteins. Inherited liver diseases are logical disease targets but several studies have also uncovered the opportunity to treat non-Mendelian diseases by liver-directed gene therapy. Expressing specific genes into hepatocytes can induce immune tolerance towards antigens that may be exploited for treatment of the deleterious consequences of immune response (e.g., inhibitor formation in hemophilias) or autoimmune disorders [18,19,20]. For example, hepatic expression of a brain protein has been shown to be protective against neuroinflammation in a mouse model of multiple sclerosis [21]. 

Several examples of liver-directed gene therapy using HDAd in monogenic disease animal models have clearly shown long term transgene expression and phenotypic correction in the absence of chronic toxicity, thus supporting the potential of HDAd for clinical applications [22,23,24,25]. Importantly, these results have also been recapitulated in clinically relevant large animal models [26,27,28,29,30] in which multi-year transgene expression has been shown [31,32]. A major factor limiting the use of these vectors in the clinic is the acute toxicity they elicit when injected systemically at high doses. The toxic response elicited by intravenously injected FGAd or multiply deleted Ad is biphasic: transduction by these early generation Ad vectors causes chronic toxicity due to viral gene expression from the vector backbone (late phase) and also results in acute toxicity (early phase). The acute response occurs within hours after vector administration and presents as a “cytokine storm” with rapid and massive elevations of serum pro-inflammatory cytokines consistent with activation of the innate inflammatory immune response. The activation of this acute response and its severity is dose-dependent [33,34,35,36], lasts for 24–48 h post-injection, and is independent of viral gene expression [37]. Indeed, the death of a partial ornithine transcarbamylase (OTC)-deficient patient, who developed a systemic inflammatory response syndrome, disseminated intravascular coagulation and multi-organ failure, was attributed to the acute toxicity from intravascular injection of a second generation (E1- and E4-deleted) Ad vector [38]. Although HDAd vectors do not cause the late phase of toxicity because they are devoid of viral genes, they can still elicit the early phase of toxicity [37,39]. 

The activation of the acute inflammatory response by systemic Ad injection is multifactorial and is observed in both rodents and nonhuman primates given comparable (on a per kg basis) systemic high doses of Ad vectors. However, mice are much more tolerant than nonhuman primates to high vector doses [34,35,40]. Differences in the innate immunity, interactions with blood cells, and hepatic microarchitecture might all contribute to the differences in the severity of the responses between species and highlight the limitations of rodents as model for investigation of the acute toxicity. 

**Figure 1:**
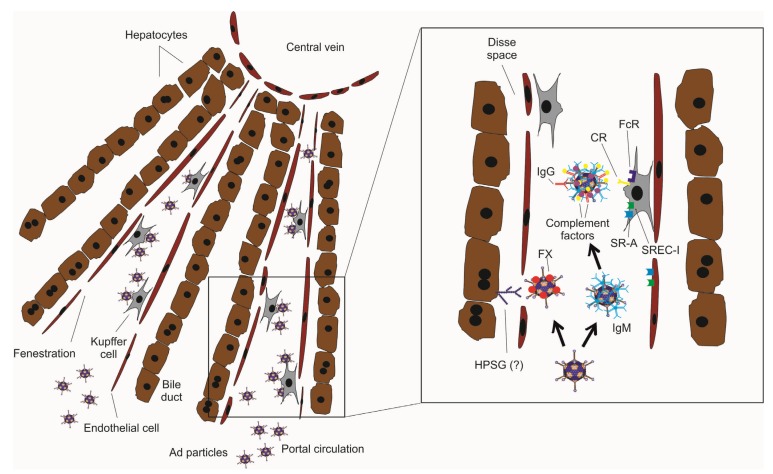
Schematic representation of the liver microarchitecture and the hurdles to efficient Ad vector-mediated hepatocyte gene therapy. Systemic delivery of Ad vector particles is hampered by binding of plasma proteins, Kupffer cell uptake, and limited permeability of endothelial cells. In the bloodstream, coagulation factor X (FX) binds with high affinity to the Ad capsid and protects from IgM and complement binding. Complement receptors bind to complement proteins bound either directly or indirectly to Ad particles. Vector particles osponized by pentameric IgM, monomeric IgG, or complement factors are recognized by different receptors (FcR, CR) on Kupffer cells. Kupffer cells and endothelial cells also express SR-A and SREC-I that bind Ad5 particles based on negative-charge interactions. The size of liver sinusoidal fenestrations affects the efficiency of Ad-mediated hepatocyte transduction. Abbreviations: HPSG, heparan sulfate proteoglycans; FX, factor X; FcR, Fc-receptor; SR-A, scavenger receptor-A; SREC-I, scavenger receptor expressed by endothelial cells 1; CR, complement receptors.

In recent years, new and important knowledge has been gained on Ad-host interactions and their role in activation of the innate immunity. According to the early model of the 1990s, Ad5 infection is dependent upon receptors for attachment (the coxsackie and adenovirus receptor, CAR) and entry (α_v_ integrins) [41,42,43]. While this mechanism is still valid for *in vitro* infection, it does not apply to *in vivo* infection, at least in the liver. Ad5-mediated hepatocyte transduction occurs independently of viral association with CAR and integrins [44,45]. Following intravascular injection, Ad particles are attacked by plasma proteins that have profound effects on vector tropism and biodistribution. In the bloodstream, coagulation factor X (FX) and VII (FVII) bind to Ad particles [46,47,48,49]. Binding to FX occurs with extremely high affinity and might function as a bridge facilitating the attachment of Ad5 to cells: the γ-carboxyglutamic acid domain of FX binds to Ad5 hexon protein and the serine protease domain of FX binds cell-surface heparan sulfate proteoglycans [50] (Figure 1). Notably, this interaction is also involved in hepatocyte gene transfer in nonhuman primates [51]. Electron cryomicroscopy studies have shown that FX binds within cavities formed by trimeric hexon proteins and interacts with Ad5 hexon hypervariable regions [52]. FX is required for Ad5 vectors to transduce hepatocytes *in vivo*: mutations in the hexon protein or FX ablation by warfarin treatment [53,54,55] both resulted in hepatocyte de-targeting and negligible hepatocyte transduction [46]. In contrast to species C serotypes Ad5 and Ad2, species B Ad35 and species D Ad26 have weak-to-no binding to FX and do not transduce efficiently the liver [56]. 

Within the bloodstream, Ad particles can activate the complement cascade independently from antibodies [57,58] and directly bind complement factors that neutralize Ad infectivity by interfering with binding to cells [48,59]. Interestingly, FX protects Ad5 from complement and allows efficient hepatocyte transduction [59] (Figure 1). Ablation of FX-Ad binding had no effect on liver transduction in mice lacking antibodies or complement components, thus questioning the hypothesis that FX is required for Ad5 binding to hepatocytes *in vivo* [59]. Based on these recent data, engineering non-FX-binding Ad vectors would not be effective for liver de-targeting as previously proposed [60], because such vectors could be exposed to increased attack by complement and may therefore have suboptimal transduction efficiency. Following macrophage internalization of FX decorated Ad particles, intracellular FX triggers activation of innate immunity via TLR4/NFKB pathway and thus, plays a role in the acute toxicity [61]. Besides TLR4 [61], other Toll-like Receptors (TLRs) are involved in Ad induced innate response, including TLR9 that senses vector dsDNA [62,63], TLR3 [64], and TLR2 [65,66].

Following intravenous Ad injection, there is a nonlinear dose response to hepatic transduction, with low doses yielding very low to undetectable levels of transgene expression, but with higher doses resulting in disproportionately high levels of transgene expression. Kupffer cells that express multiple receptors for antibodies and complement are responsible for this nonlinear dose response because they avidly sequester bloodborne Ad particles [67,68] (Figure 1). Vector uptake by Kupffer cells is detrimental for therapeutic applications because it reduces hepatocyte infection at clinically safer vector doses and by limiting tumor viral uptake it decreases the efficacy of intravenously injected Ad in patients with metastatic cancers [69,70,71]. Natural or preexisting neutralizing antibodies are involved in vector clearance by Kupffer cells [59,72] through opsonization of vector particles that enhance Fc-receptor mediated vector uptake. Antibodies can also bind indirectly to viral particles through binding to complement factor C3 [73]. Antibody-virus complexes activate the classical complement proteins C1, C2, and C4 [57,59]. Complement activation result in covalent binding of C3 fragments to viral capsid and Ad particle uptake by Kupffer cells via Complement Receptor Ig-superfamily (CRIg) that regulate death of these cells in the liver [74] (Figure 1). 

Following uptake of bloodborne Ad particles, Kupffer cells undergo rapid pro-inflammatory necrotic death that is controlled by interferon-regulatory factor 3 (IRF3) [75,76,77,78]. Ad uptake by Kupffer cells and their necrosis appear to play a protective role and may represent a defensive suicide strategy preventing disseminated virus infection [61]. Consequently, for the host that lacks this macrophage population, even a sublethal virus infection may be detrimental for survival. Mice depleted of tissue macrophages by clodronate liposomes showed in fact higher virus DNA burden, greater hepatotoxicity, and increased lethality [61]. Moreover, intravenously injected Ad5 causes a rapid hemodynamic response presenting with hypotension, hemoconcentration, tissue edema, and vasocongestion [77,79] that is dependent, at least in part, on upregulation in macrophages of platelet-activating factor (PAF), a known shock inducer lipid signaling molecule [79]. 

The observation that administration of polyinosine, as well as other polyanionic ligands, into mice prior to Ad intravenous injection drastically reduces Ad accumulation in Kupffer cells and increases hepatocyte gene transfer [73,80], has led to the recognition of scavenger receptor-A (SR-A) and scavenger receptor expressed on endothelial cells type I (SREC-I) on both Kupffer cells and endothelial cells as mediators of Ad vector uptake [81,82,83] (Figure 1).

The presence of endothelium fenestrations in the liver permits transduction of hepatocytes by Ad vectors whereas other tissues that lack such fenestrations are poorly transduced [84,85]. Nevertheless, several studies in animal models suggest that the diameter of liver endothelial fenestrations has species- and strain-specific differences in size (e.g., ~141 nm in C57BL/6 mice, 124 nm in Dutch Belt rabbits, and ~103 nm in NZW rabbits) and plays an important role in the efficiency of Ad-mediated hepatocyte transduction [86,87,88]. Ad5 particles have a diameter of 93 nm with protruding fibers of 30 nm [87] whereas the diameter of human liver fenestration is ~107 nm and thus, the relative smaller size of liver fenestrations may represent an obstacle for hepatocyte transduction in humans [88].

Systemic administration of high doses of Ad vectors likely results in widespread transduction of a large number of various extrahepatic cell types (e.g., blood cells, endothelium, spleen, and lung) which are also important barriers to efficient hepatocyte transduction. Over 90% of Ad vectors bind to human erythrocytes *ex vivo* [89] through the CAR that is expressed on erythrocytes from humans but not from mice or rhesus macaques [90]. Furthermore, erythrocytes from humans but not from mice bear the complement receptor 1 (CR1) which binds Ad5 in the presence of antibodies and complement [90]. Although the liver takes up more vector than any other organ following systemic injection, the total amount of vector, on a vector genome copy number per μg DNA basis, is abundantly distributed throughout the body in mice [35], in nonhuman primates [34,91], and in a human patient [38].

## 3. Strategies to Improve HDAd Therapeutic Index

Given the potential of HDAd vectors for gene therapy, several groups have investigated various strategies to overcome the obstacle of acute toxicity. Because the severity of the acute response is dose-dependent and appears to correlate with extrahepatic systemic vector dissemination, one of these strategies aimed at targeting the vector to the liver thereby allowing the use of lower vector doses. This has been accomplished using physical approaches aiming at preferential hepatocyte transduction. One such strategy was to deliver the HDAd into surgically isolated livers to limit systemic exposure and resulted in greater hepatocyte transduction compared to intravenous injection and no chronic toxicity in nonhuman primates [27]. A follow-up of this method reported that transgene expression had persisted for up to 7 years without long-term adverse effects [31]. Over this long observation period, that is more than half the life span of most captive baboons that were used in the study [92], transgene expression showed a gradual decline that is consistent with the nuclear status of HDAd vector genome and the low hepatocyte turn-over. Within the nuclei of the transduced cells, the HDAd genome is episomal and it is lost during cell division [93]. Notably, the frequency of HDAd genome integration is low [94] and thus, the likelihood that Ad vector integration would result in the inactivation of genes or activation of proto-oncogenes is relatively low. 

To overcome the limitations due to the invasiveness of the surgical delivery method, a minimally invasive, percutaneous balloon occlusion catheter-based method was then developed to achieve preferential hepatocyte transduction. In this method, a sausage-shaped balloon is inflated in the inferior vena cava to occlude the hepatic venous outflow and the HDAd is injected directly into the liver via the hepatic artery. This vector delivery method resulted in high levels of transgene expression compared to peripheral intravenous injection in nonhuman primates using clinically relevant low doses [26,29]. Furthermore, this high-level transgene expression persisted for several years in the absence of chronic toxicity at levels that would be therapeutic, at least for hemophilia B [29,31,95]. 

In another approach, effective dose reduction was achieved by direct injections of HDAd into the liver parenchyma that resulted in improved efficacy and reduced toxicity. Direct vector injections into liver parenchyma is a relatively simple and flexible technique that is similar to the procedure accomplished routinely for liver biopsies and is performed in humans for treatment of hepatic metastases [96]. However, the absolute number of hepatocytes that can be transduced by this method is limited [97] and thus, this approach is unsuitable for diseases requiring a large number of transduced hepatocytes to obtain clinically relevant benefits. Nevertheless, it remains attractive for liver diseases affecting discrete hepatic regions, such as liver metastases, for delivery of secreted therapeutic genes, and for disorders requiring a small percentage of hepatocyte correction to obtain phenotypic improvements, such as Crigler–Najjar syndrome [97,98]. 

Kupffer cell depletion by pre-treatment with gadolinium chloride, dichloromethylene biphosponate, or clodronate lyposomes prior to vector injection result in enhanced hepatocyte transduction [68,99,100,101], but applications of these agents is limited by their side effects [100,102,103,104]. Inhibition of SR-A and SREC-I mediated uptake of Ad vectors by Kupffer and endothelial cells results in increased hepatocyte transduction and is another attractive option to increase the vector therapeutic index [83].

Genetic or chemical modifications or a combination of these changes to the viral capsid is another strategy that has been investigated to evade Kupffer cell uptake. Given that Ad-Kupffer cell interaction is mediated by the hypervariable loops of the virus hexon protein, Ad vectors have been genetically modified to disrupt such interactions. A chimeric vector in which the hypervariable region of Ad5 is replaced with that of Ad6 showed reduced Kupffer cell uptake, higher levels of liver transduction, and reduced hepatotoxicity compared to Ad5 vector [105]. The hypervariable regions of Ad5 have also been modified with cysteine residues to enable blocking of these sites with polyethylene glycol (PEG). After intravenous injection, targeted PEGylation of hypervariable regions increased vector-mediated hepatocyte transduction [82]. Site-specific coupling of 5K PEG or transferrin to hexon capsid protein of Ad vectors also improved liver transduction [106,107] whereas decoration of viral particles with higher molecular weight 20K PEG de-targeted vectors from both hepatocytes and Kupffer cells [107,108]. Nevertheless, as explained above, these modifications might also make the vector more prone to complement binding and thus less effective for liver de-targeting [59]. 

In addition, “masking” the viral capsid has also been reported to attenuate the severity of the innate inflammatory response. Systemic administration of non-specific PEGylated Ad into mice resulted in 50%–70% reduction of serum IL-6 compared to unPEGylated vector without compromising hepatic transduction [109,110,111]. Although the above studies showed that hepatic transduction in mice was not affected by PEGylated HDAd, this was not the case in nonhuman primates where hepatic transduction was reduced [112], emphasizing that caution should be taken in extrapolating results from rodents to larger animals and humans.

## 4. Human Applications of HDAd

There was a single case of intravascular administration of an HDAd vector into a human patient; however, this study has not been published in a peer-reviewed format and much of the details are not available. From what is known, a hemophilia A patient received by intravenous injection 4.3 × 10^11^ vp/kg of a HDAd expressing factor VIII (FVIII) [113]. This subject developed grade 3 liver toxicity, marked increase in IL-6, thrombocytopenia, and laboratory signs of disseminated intravascular coagulopathy, but all these values returned to baseline by day 19 post-infusion. Unfortunately, no evidence of FVIII expression was detected [113]. 

HDAd has recently been used in an *ex vivo* clinical trial to treat anemia in patients with chronic kidney failure [114]. In this phase I-II study, dermal fibroblasts were removed from the skin of patients, transduced *ex vivo* with an HDAd expressing erythropoietin (EPO), and implanted under local anesthesia in the subcutaneous tissue in an autologous manner. To achieve the pre-determined blood levels of EPO, a precise number of HDAd transduced cells was implanted. There were no adverse events in this trial and hemoglobin levels were sustained for up to one year after a single treatment with the HDAd transduced cells [114]. This trial also demonstrates that HDAd can be manufactured under cGMP for a human clinical trial.

## 5. Conclusions

HDAd are attractive vectors for gene therapy particularly given their ability to drive long-term transgene expression with no chronic toxicity and with low risk of insertional carcinogenesis. To date, in small and large models, including nonhuman primates, HDAd transduced hepatocytes (as well as all other target cell types examined) are not eliminated by the immune system and result in multi-year transgene expression. Nevertheless, whether this holds true for humans is not known at the present time, particularly in consideration of the outcomes of the AAV clinical trials for hemophilia B that resulted in a CTL immune response against AAV-transduced hepatocytes [115,116].

The dose-dependent activation of the innate inflammatory response by viral capsids remains an important concern for those applications requiring systemic intravenous injection of high vector dose to achieve clinically relevant benefits. A significant amount of data on vector biodistribution, toxicity, and efficacy has been generated through studies performed in small animal models and clearly these studies have led to increased knowledge in the field. However, the translational relevance of several of these findings requires further validation in larger animal models that more closely predict human outcomes. The multitude of host factors interacting with Ad particles has highlighted a series of unforeseen and intriguing mechanisms that illustrate the complexity of Ad vector-host interactions. Importantly, these studies have identified potential molecules that could be targeted to avoid vector scavenging and degradation by macrophages with the goal of achieving efficient gene transfer to hepatocytes or tumors. Pharmacological approaches directed towards these targets in combination with advancements in vector targeting or de-targeting, or with physical methods, such as balloon catheter-assisted delivery, would result in more efficient and safer gene delivery with potential for clinical applications. 

## References

[1] Gene Therapy Clinical Trials Worldwide. http://www.wiley.com/legacy/wileychi/genmed/clinical/.

[2] Shenk T. (1996). Adenoviridae: The Viruses and Their Replication. In Fields Viology, BN Fields, DM Knipe, PM.

[3] Graham F.L., Smiley J., Russell W.C., Nairn R. (1977). Characteristics of a human cell line transformed by DNA from human adenovirus type 5. J. Gene Virol..

[4] Morral N., OʼNeal W., Zhou H., Langston C., Beaudet A. (1997). Immune responses to reporter proteins and high viral dose limit duration of expression with adenoviral vectors: Comparison of E2a wild type and E2a deleted vectors. Hum. Gene Ther..

[5] Brunetti-Pierri N., Ng P. (2011). Helper-dependent adenoviral vectors for liver-directed gene therapy. Hum. Mol. Genet..

[6] Parks R.J., Chen L., Anton M., Sankar U., Rudnicki M.A., Graham F.L. (1996). A helper-dependent adenovirus vector system: Removal of helper virus by Cre-mediated excision of the viral packaging signal. Proc. Natl. Acad. Sci. USA.

[7] Palmer D., Ng P. (2003). Improved system for helper-dependent adenoviral vector production. Mol. Ther..

[8] Palmer D., Ng P. (2007). Methods for the Production and Characterization of Helper-Dependent Adenoviral Vectors.

[9] Palmer D., Ng P. (2008). Methods for the preparation of helper-dependent adenoviral vectors. Methods Mol. Med..

[10] Immonen A., Vapalahti M., Tyynela K., Hurskainen H., Sandmair A., Vanninen R., Langford G., Murray N., Yla-Herttuala S. (2004). AdvHSV-tk gene therapy with intravenous ganciclovir improves survival in human malignant glioma: A randomised, controlled study. Mol. Ther..

[11] Kirn D. (2001). Clinical research results with dl1520 (Onyx-015), a replication-selective adenovirus for the treatment of cancer: What have we learned?. Gene Ther..

[12] Peng Z. (2005). Current status of gendicine in China: Recombinant human Ad-p53 agent for treatment of cancers. Hum. Gene Ther..

[13] Freytag S.O., Stricker H., Movsas B., Kim J.H. (2007). Prostate cancer gene therapy clinical trials. Mol. Ther..

[14] Lasaro M.O., Ertl H.C. (2009). New insights on adenovirus as vaccine vectors. Mol. Ther..

[15] Farzad L.M., Suzuki M. (2014). Feasibility of applying helper-dependent adenoviral vectors for cancer immunotherapy. Biomedicines.

[16] Brunetti-Pierri N., Ng P. (2006). Progress towards the clinical application of helper-dependent adenoviral vectors for liver and lung gene therapy. Curr. Opin. Mol. Ther..

[17] Brunetti-Pierri N., Ng P. (2009). Progress towards liver and lung-directed gene therapy with helper-dependent adenoviral vectors. Curr. Gene Ther..

[18] Brown B.D., Cantore A., Annoni A., Sergi L.S., Lombardo A., Della Valle P., D’Angelo A., Naldini L. (2007). A microRNA-regulated lentiviral vector mediates stable correction of hemophilia B mice. Blood.

[19] Cerullo V., McCormack W., Seiler M., Mane V., Cela R., Clarke C., Rodgers J.R., Lee B. (2007). Antigen-specific tolerance of human alpha1-antitrypsin induced by helper-dependent adenovirus. Hum. Gene Ther..

[20] Mingozzi F., Liu Y.L., Dobrzynski E., Kaufhold A., Liu J.H., Wang Y., Arruda V.R., High K.A., Herzog R.W. (2003). Induction of immune tolerance to coagulation factor IX antigen by *in vivo* hepatic gene transfer. J. Clin. Invest..

[21] Luth S., Huber S., Schramm C., Buch T., Zander S., Stadelmann C., Bruck W., Wraith D.C., Herkel J., Lohse A.W. (2008). Ectopic expression of neural autoantigen in mouse liver suppresses experimental autoimmune neuroinflammation by inducing antigen-specific Tregs. J. Clin. Invest..

[22] Kim I.H., Jozkowicz A., Piedra P.A., Oka K., Chan L. (2001). Lifetime correction of genetic deficiency in mice with a single injection of helper-dependent adenoviral vector. Proc. Natl. Acad. Sci. USA.

[23] Toietta G., Mane V.P., Norona W.S., Finegold M.J., Ng P., McDonagh A.F., Beaudet A.L., Lee B. (2005). Lifelong elimination of hyperbilirubinemia in the Gunn rat with a single injection of helper-dependent adenoviral vector. Proc. Natl. Acad. Sci. USA.

[24] Dimmock D., Brunetti-Pierri N., Palmer D., Beaudet A., Ng P. (2011). Correction of hyperbilirubinemia in gunn rats using clinically relevant low doses of helper-dependent adenoviral vectors. Hum. Gene Ther..

[25] Brunetti-Pierri N., Grove N.C., Zuo Y., Edwards R., Palmer D., Cerullo V., Teruya J., Ng P. (2009). Bioengineered factor IX molecules with increased catalytic activity improve the therapeutic index of gene therapy vectors for hemophilia B. Hum. Gene Ther..

[26] Brunetti-Pierri N., Stapleton G.E., Palmer D.J., Zuo Y., Mane V.P., Finegold M.J., Beaudet A.L., Leland M.M., Mullins C.E., Ng P. (2007). Pseudo-hydrodynamic delivery of helper-dependent adenoviral vectors into non-human primates for liver-directed gene therapy. Mol. Ther..

[27] Brunetti-Pierri N., Ng T., Iannitti D.A., Palmer D.J., Beaudet A.L., Finegold M.J., Carey K.D., Cioffi W.G., Ng P. (2006). Improved hepatic transduction, reduced systemic vector dissemination, and long-term transgene expression by delivering helper-dependent adenoviral vectors into the surgically isolated liver of nonhuman primates. Hum. Gene Ther..

[28] Morral N., O’Neal W., Rice K., Leland M., Kaplan J., Piedra P.A., Zhou H., Parks R.J., Velji R., Aguilar-Cordova E. (1999). Administration of helper-dependent adenoviral vectors and sequential delivery of different vector serotype for long-term liver-directed gene transfer in baboons. Proc. Natl. Acad. Sci. USA.

[29] Brunetti-Pierri N., Stapleton G.E., Law M., Breinholt J., Palmer D.J., Zuo Y., Grove N.C., Finegold M.J., Rice K., Beaudet A.L. (2009). Efficient, long-term hepatic gene transfer using clinically relevant HDAd doses by balloon occlusion catheter delivery in nonhuman primates. Mol. Ther..

[30] Brunetti-Pierri N., Nichols T.C., McCorquodale S., Merricks E., Palmer D.J., Beaudet A.L., Ng P. (2005). Sustained phenotypic correction of canine hemophilia B after systemic administration of helper-dependent adenoviral vector. Hum. Gene Ther..

[31] Brunetti-Pierri N., Ng T., Iannitti D., Cioffi W., Stapleton G., Law M., Breinholt J., Palmer D., Grove N., Rice K. (2013). Transgene expression up to 7 years in nonhuman primates following hepatic transduction with helper-dependent adenoviral vectors. Hum. Gene Ther..

[32] Crane B., Luo X., Demaster A., Williams K.D., Kozink D.M., Zhang P., Brown T.T., Pinto C.R., Oka K., Sun F. (2012). Rescue administration of a helper-dependent adenovirus vector with long-term efficacy in dogs with glycogen storage disease type Ia. Gene Ther..

[33] Muruve D.A., Barnes M.J., Stillman I.E., Libermann T.A. (1999). Adenoviral gene therapy leads to rapid induction of multiple chemokines and acute neutrophil-dependent hepatic injury *in vivo*. Hum. Gene Ther..

[34] Schnell M.A., Zhang Y., Tazelaar J., Gao G.P., Yu Q.C., Qian R., Chen S.J., Varnavski A.N., LeClair C., Raper S.E. (2001). Activation of innate immunity in nonhuman primates following intraportal administration of adenoviral vectors. Mol. Ther..

[35] Zhang Y., Chirmule N., Gao G.P., Qian R., Croyle M., Joshi B., Tazelaar J., Wilson J.M. (2001). Acute cytokine response to systemic adenoviral vectors in mice is mediated by dendritic cells and macrophages. Mol. Ther..

[36] Liu Q., Muruve D.A. (2003). Molecular basis of the inflammatory response to adenovirus vectors. Gene Ther..

[37] Brunetti-Pierri N., Palmer D.J., Beaudet A.L., Carey K.D., Finegold M., Ng P. (2004). Acute toxicity after high-dose systemic injection of helper-dependent adenoviral vectors into nonhuman primates. Hum. Gene Ther..

[38] Raper S.E., Chirmule N., Lee F.S., Wivel N.A., Bagg A., Gao G.P., Wilson J.M., Batshaw M.L. (2003). Fatal systemic inflammatory response syndrome in a ornithine transcarbamylase deficient patient following adenoviral gene transfer. Mol. Genet. Metab..

[39] Muruve D.A., Cotter M.J., Zaiss A.K., White L.R., Liu Q., Chan T., Clark S.A., Ross P.J., Meulenbroek R.A., Maelandsmo G.M. (2004). Helper-dependent adenovirus vectors elicit intact innate but attenuated adaptive host immune responses *in vivo*. J. Virol..

[40] Morral N., O’Neal W.K., Rice K., Leland M.M., Piedra P.A., Aguilar-Cordova E., Carey K.D., Beaudet A.L., Langston C. (2002). Lethal toxicity, severe endothelial injury, and a threshold effect with high doses of an adenoviral vector in baboons. Hum. Gene Ther..

[41] Bergelson J.M., Cunningham J.A., Droguett G., Kurt-Jones E.A., Krithivas A., Hong J.S., Horwitz M.S., Crowell R.L., Finberg R.W. (1997). Isolation of a common receptor for Coxsackie B viruses and adenoviruses 2 and 5. Science.

[42] Tomko R.P., Xu R., Philipson L. (1997). HCAR and MCAR: The human and mouse cellular receptors for subgroup C adenoviruses and group B coxsackieviruses. Proc. Natl. Acad. Sci. USA.

[43] Wickham T.J., Mathias P., Cheresh D.A., Nemerow G.R. (1993). Integrins alpha v beta 3 and alpha v beta 5 promote adenovirus internalization but not virus attachment. Cell.

[44] Martin K., Brie A., Saulnier P., Perricaudet M., Yeh P., Vigne E. (2003). Simultaneous CAR- and alpha V integrin-binding ablation fails to reduce Ad5 liver tropism. Mol. Ther..

[45] Alemany R., Curiel D.T. (2001). CAR-binding ablation does not change biodistribution and toxicity of adenoviral vectors. Gene Ther..

[46] Parker A.L., Waddington S.N., Nicol C.G., Shayakhmetov D.M., Buckley S.M., Denby L., Kemball-Cook G., Ni S., Lieber A., McVey J.H. (2006). Multiple vitamin K-dependent coagulation zymogens promote adenovirus-mediated gene delivery to hepatocytes. Blood.

[47] Parker A.L., McVey J.H., Doctor J.H., Lopez-Franco O., Waddington S.N., Havenga M.J., Nicklin S.A., Baker A.H. (2007). Influence of coagulation factor zymogens on the infectivity of adenoviruses pseudotyped with fibers from subgroup D. J. Virol..

[48] Shayakhmetov D.M., Gaggar A., Ni S., Li Z.Y., Lieber A. (2005). Adenovirus binding to blood factors results in liver cell infection and hepatotoxicity. J. Virol..

[49] Waddington S.N., Parker A.L., Havenga M., Nicklin S.A., Buckley S.M., McVey J.H., Baker A.H. (2007). Targeting of adenovirus serotype 5 (Ad5) and 5/47 pseudotyped vectors *in vivo*: Fundamental involvement of coagulation factors and redundancy of CAR binding by Ad5. J. Virol..

[50] Duffy M.R., Bradshaw A.C., Parker A.L., McVey J.H., Baker A.H. (2011). A cluster of basic amino acids in the factor X serine protease mediates surface attachment of adenovirus/FX complexes. J. Virol..

[51] Alba R., Bradshaw A.C., Mestre-Frances N., Verdier J.M., Henaff D., Baker A.H. (2012). Coagulation factor X mediates adenovirus type 5 liver gene transfer in non-human primates (Microcebus murinus). Gene Ther..

[52] Waddington S.N., McVey J.H., Bhella D., Parker A.L., Barker K., Atoda H., Pink R., Buckley S.M., Greig J.A., Denby L. (2008). Adenovirus serotype 5 hexon mediates liver gene transfer. Cell.

[53] Di Paolo N.C., van Rooijen N., Shayakhmetov D.M. (2009). Redundant and synergistic mechanisms control the sequestration of blood-born adenovirus in the liver. Mol. Ther..

[54] Bradshaw A.C., Coughlan L., Miller A.M., Alba R., van Rooijen N., Nicklin S.A., Baker A.H. (2012). Biodistribution and inflammatory profiles of novel penton and hexon double-mutant serotype 5 adenoviruses. J. Control. Release.

[55] Alba R., Bradshaw A.C., Coughlan L., Denby L., McDonald R.A., Waddington S.N., Buckley S.M., Greig J.A., Parker A.L., Miller A.M. (2010). Biodistribution and retargeting of FX-binding ablated adenovirus serotype 5 vectors. Blood.

[56] Alba R., Bradshaw A.C., Parker A.L., Bhella D., Waddington S.N., Nicklin S.A., van Rooijen N., Custers J., Goudsmit J., Barouch D.H. (2009). Identification of coagulation factor (F)X binding sites on the adenovirus serotype 5 hexon: Effect of mutagenesis on FX interactions and gene transfer. Blood.

[57] Tian J., Xu Z., Smith J.S., Hofherr S.E., Barry M.A., Byrnes A.P. (2009). Adenovirus activates complement by distinctly different mechanisms *in vitro* and *in vivo*: Indirect complement activation by virions *in vivo*. J. Virol..

[58] Jiang H., Wang Z., Serra D., Frank M.M., Amalfitano A. (2004). Recombinant adenovirus vectors activate the alternative complement pathway, leading to the binding of human complement protein C3 independent of anti-ad antibodies. Mol. Ther..

[59] Xu Z., Qiu Q., Tian J., Smith J.S., Conenello G.M., Morita T., Byrnes A.P. (2013). Coagulation factor X shields adenovirus type 5 from attack by natural antibodies and complement. Nat. Med..

[60] Coughlan L., Alba R., Parker A.L., Bradshaw A.C., McNeish I.A., Nicklin S.A., Baker A.H. (2010). Tropism-modification strategies for targeted gene delivery using adenoviral vectors. Viruses.

[61] Doronin K., Flatt J.W., Di Paolo N.C., Khare R., Kalyuzhniy O., Acchione M., Sumida J.P., Ohto U., Shimizu T., Akashi-Takamura S. (2012). Coagulation factor X activates innate immunity to human species C adenovirus. Science.

[62] Cerullo V., Seiler M.P., Mane V., Brunetti-Pierri N., Clarke C., Bertin T.K., Rodgers J.R., Lee B. (2007). Toll-like receptor 9 triggers an innate immune response to helper-dependent adenoviral vectors. Mol. Ther..

[63] Basner-Tschakarjan E., Gaffal E., O’Keeffe M., Tormo D., Limmer A., Wagner H., Hochrein H., Tuting T. (2006). Adenovirus efficiently transduces plasmacytoid dendritic cells resulting in TLR9-dependent maturation and IFN-alpha production. J. Gene Med..

[64] Appledorn D.M., Patial S., Godbehere S., Parameswaran N., Amalfitano A. (2009). TRIF, and TRIF-interacting TLRs differentially modulate several adenovirus vector-induced immune responses. J. Innate Immun..

[65] Appledorn D.M., Patial S., McBride A., Godbehere S., van Rooijen N., Parameswaran N., Amalfitano A. (2008). Adenovirus vector-induced innate inflammatory mediators, MAPK signaling, as well as adaptive immune responses are dependent upon both TLR2 and TLR9 *in vivo*. J. Immunol..

[66] Suzuki M., Cerullo V., Bertin T.K., Cela R., Clarke C., Guenther M., Brunetti-Pierri N., Lee B. (2010). MyD88-dependent silencing of transgene expression during the innate and adaptive immune response to helper-dependent adenovirus. Hum. Gene Ther..

[67] Tao N., Gao G.P., Parr M., Johnston J., Baradet T., Wilson J.M., Barsoum J., Fawell S.E. (2001). Sequestration of adenoviral vector by Kupffer cells leads to a nonlinear dose response of transduction in liver. Mol. Ther..

[68] Schiedner G., Hertel S., Johnston M., Dries V., van Rooijen N., Kochanek S. (2003). Selective depletion or blockade of Kupffer cells leads to enhanced and prolonged hepatic transgene expression using high-capacity adenoviral vectors. Mol. Ther..

[69] Nemunaitis J., Cunningham C., Buchanan A., Blackburn A., Edelman G., Maples P., Netto G., Tong A., Randlev B., Olson S. (2001). Intravenous infusion of a replication-selective adenovirus (ONYX-015) in cancer patients: Safety, feasibility and biological activity. Gene Ther..

[70] Small E.J., Carducci M.A., Burke J.M., Rodriguez R., Fong L., van Ummersen L., Yu D.C., Aimi J., Ando D., Working P. (2006). A phase I trial of intravenous CG7870, a replication-selective, prostate-specific antigen-targeted oncolytic adenovirus, for the treatment of hormone-refractory, metastatic prostate cancer. Mol. Ther..

[71] Nemunaitis J., Senzer N., Sarmiento S., Zhang Y.A., Arzaga R., Sands B., Maples P., Tong A.W. (2007). A phase I trial of intravenous infusion of ONYX-015 and enbrel in solid tumor patients. Cancer Gene Ther..

[72] Khare R., Hillestad M.L., Xu Z., Byrnes A.P., Barry M.A. (2013). Circulating antibodies and macrophages as modulators of adenovirus pharmacology. J. Virol..

[73] Xu Z., Tian J., Smith J.S., Byrnes A.P. (2008). Clearance of adenovirus by Kupffer cells is mediated by scavenger receptors, natural antibodies, and complement. J. Virol..

[74] He J.Q., Katschke K.J., Gribling P., Suto E., Lee W.P., Diehl L., Eastham-Anderson J., Ponakala A., Komuves L., Egen J.G. (2013). CRIg mediates early Kupffer cell responses to adenovirus. J. Leukoc. Biol..

[75] Di Paolo N.C., Doronin K., Baldwin L.K., Papayannopoulou T., Shayakhmetov D.M. (2013). The transcription factor IRF3 triggers “defensive suicide” necrosis in response to viral and bacterial pathogens. Cell Rep..

[76] Smith J.S., Xu Z., Tian J., Stevenson S.C., Byrnes A.P. (2008). Interaction of systemically delivered adenovirus vectors with Kupffer cells in mouse liver. Hum. Gene Ther..

[77] Schiedner G., Bloch W., Hertel S., Johnston M., Molojavyi A., Dries V., Varga G., van Rooijen N., Kochanek S. (2003). A hemodynamic response to intravenous adenovirus vector particles is caused by systemic Kupffer cell-mediated activation of endothelial cells. Hum. Gene Ther..

[78] Nociari M., Ocheretina O., Schoggins J.W., Falck-Pedersen E. (2007). Sensing infection by adenovirus: Toll-like receptor-independent viral DNA recognition signals activation of the interferon regulatory factor 3 master regulator. J. Virol..

[79] Xu Z., Smith J.S., Tian J., Byrnes A.P. (2010). Induction of shock after intravenous injection of adenovirus vectors: A critical role for platelet-activating factor. Mol. Ther..

[80] Haisma H.J., Kamps J.A., Kamps G.K., Plantinga J.A., Rots M.G., Bellu A.R. (2008). Polyinosinic acid enhances delivery of adenovirus vectors *in vivo* by preventing sequestration in liver macrophages. J. Gene Virol..

[81] Haisma H.J., Boesjes M., Beerens A.M., van der Strate B.W., Curiel D.T., Pluddemann A., Gordon S., Bellu A.R. (2009). Scavenger receptor A: A new route for adenovirus 5. Mol. Pharm..

[82] Khare R., Reddy V.S., Nemerow G.R., Barry M.A. (2012). Identification of adenovirus serotype 5 hexon regions that interact with scavenger receptors. J. Virol..

[83] Piccolo P., Vetrini F., Mithbaokar P., Grove N.C., Bertin T., Palmer D., Ng P., Brunetti-Pierri N. (2013). SR-A and SREC-I are kupffer and endothelial cell receptors for helper-dependent adenoviral vectors. Mol. Ther..

[84] Ganesan L.P., Mohanty S., Kim J., Clark K.R., Robinson J.M., Anderson C.L. (2011). Rapid and efficient clearance of blood-borne virus by liver sinusoidal endothelium. PLoS Pathog..

[85] Yu Q., Que L.G., Rockey D.C. (2002). Adenovirus-mediated gene transfer to nonparenchymal cells in normal and injured liver. Am. J. Physiol. Gastrointest. Liver Physiol..

[86] Lievens J., Snoeys J., Vekemans K., van Linthout S., de Zanger R., Collen D., Wisse E., de Geest B. (2004). The size of sinusoidal fenestrae is a critical determinant of hepatocyte transduction after adenoviral gene transfer. Gene Ther..

[87] Snoeys J., Lievens J., Wisse E., Jacobs F., Duimel H., Collen D., Frederik P., de Geest B. (2007). Species differences in transgene DNA uptake in hepatocytes after adenoviral transfer correlate with the size of endothelial fenestrae. Gene Ther..

[88] Wisse E., Jacobs F., Topal B., Frederik P., de Geest B. (2008). The size of endothelial fenestrae in human liver sinusoids: Implications for hepatocyte-directed gene transfer. Gene Ther..

[89] Lyons M., Onion D., Green N.K., Aslan K., Rajaratnam R., Bazan-Peregrino M., Phipps S., Hale S., Mautner V., Seymour L.W. (2006). Adenovirus type 5 interactions with human blood cells may compromise systemic delivery. Mol. Ther..

[90] Carlisle R.C., Di Y., Cerny A.M., Sonnen A.F., Sim R.B., Green N.K., Subr V., Ulbrich K., Gilbert R.J., Fisher K.D. (2009). Human erythrocytes bind and inactivate type 5 adenovirus by presenting coxsackievirus-adenovirus receptor and complement receptor 1. Blood.

[91] Varnavski A.N., Zhang Y., Schnell M., Tazelaar J., Louboutin J.P., Yu Q.C., Bagg A., Gao G.P., Wilson J.M. (2002). Preexisting immunity to adenovirus in rhesus monkeys fails to prevent vector-induced toxicity. J. Virol..

[92] Martin L.J., Mahaney M.C., Bronikowski A.M., Dee Carey K., Dyke B., Comuzzie A.G. (2002). Lifespan in captive baboons is heritable. Mech. Ageing Dev..

[93] Jager L., Ehrhardt A. (2009). Persistence of high-capacity adenoviral vectors as replication-defective monomeric genomes *in vitro* and in murine liver. Hum. Gene Ther..

[94] Stephen S.L., Montini E., Sivanandam V.G., Al-Dhalimy M., Kestler H.A., Finegold M., Grompe M., Kochanek S. (2010). Chromosomal integration of adenoviral vector DNA *in vivo*. J. Virol..

[95] Brunetti-Pierri N., Liou A., Patel P., Palmer D., Grove N., Finegold M., Piccolo P., Donnachie E., Rice K., Beaudet A. (2012). Balloon catheter delivery of helper-dependent adenoviral vector results in sustained, therapeutic hFIX expression in rhesus macaques. Mol. Ther..

[96] Sung M.W., Chen S.H., Thung S.N., Zhang D.Y., Huang T.G., Mandeli J.P., Woo S.L. (2002). Intratumoral delivery of adenovirus-mediated interleukin-12 gene in mice with metastatic cancer in the liver. Hum. Gene Ther..

[97] Pastore N., Nusco E., Piccolo P., Castaldo S., Vanikova J., Vetrini F., Palmer D.J., Vitek L., Ng P., Brunetti-Pierri N. (2013). Improved efficacy and reduced toxicity by ultrasound-guided intrahepatic injections of helper-dependent adenoviral vector in Gunn rats. Hum. Gene Ther. Methods.

[98] Brunetti-Pierri N., Lee B. (2005). Gene therapy for inborn errors of liver metabolism. Mol. Genet. Metab..

[99] Kuzmin A.I., Finegold M.J., Eisensmith R.C. (1997). Macrophage depletion increases the safety, efficacy and persistence of adenovirus-mediated gene transfer *in vivo*. Gene Ther..

[100] Wolff G., Worgall S., van Rooijen N., Song W.R., Harvey B.G., Crystal R.G. (1997). Enhancement of *in vivo* adenovirus-mediated gene transfer and expression by prior depletion of tissue macrophages in the target organ. J. Virol..

[101] Lieber A., He C.Y., Meuse L., Schowalter D., Kirillova I., Winther B., Kay M.A. (1997). The role of Kupffer cell activation and viral gene expression in early liver toxicity after infusion of recombinant adenovirus vectors. J. Virol..

[102] Ishiyama H., Sato M., Matsumura K., Sento M., Ogino K., Hobara T. (1995). Proliferation of hepatocytes and attenuation from carbon tetrachloride hepatotoxicity by gadolinium chloride in rats. Pharmacol. Toxicol..

[103] Callery M.P., Kamei T., Flye M.W. (1990). Kupffer cell blockade increases mortality during intra-abdominal sepsis despite improving systemic immunity. Arch. Surg..

[104] Salkowski C.A., Neta R., Wynn T.A., Strassmann G., van Rooijen N., Vogel S.N. (1995). Effect of liposome-mediated macrophage depletion on LPS-induced cytokine gene expression and radioprotection. J. Immunol..

[105] Khare R., May S.M., Vetrini F., Weaver E.A., Palmer D., Rosewell A., Grove N., Ng P., Barry M.A. (2011). Generation of a Kupffer cell-evading adenovirus for systemic and liver-directed gene transfer. Mol. Ther..

[106] Prill J.M., Espenlaub S., Samen U., Engler T., Schmidt E., Vetrini F., Rosewell A., Grove N., Palmer D., Ng P. (2011). Modifications of adenovirus hexon allow for either hepatocyte detargeting or targeting with potential evasion from Kupffer cells. Mol. Ther..

[107] Hofherr S.E., Shashkova E.V., Weaver E.A., Khare R., Barry M.A. (2008). Modification of adenoviral vectors with polyethylene glycol modulates *in vivo* tissue tropism and gene expression. Mol. Ther..

[108] Doronin K., Shashkova E.V., May S.M., Hofherr S.E., Barry M.A. (2009). Chemical modification with high molecular weight polyethylene glycol reduces transduction of hepatocytes and increases efficacy of intravenously delivered oncolytic adenovirus. Hum. Gene Ther..

[109] Mok H., Palmer D.J., Ng P., Barry M.A. (2005). Evaluation of polyethylene glycol modification of first-generation and helper-dependent adenoviral vectors to reduce innate immune responses. Mol. Ther..

[110] Croyle M.A., Le H.T., Linse K.D., Cerullo V., Toietta G., Beaudet A., Pastore L. (2005). PEGylated helper-dependent adenoviral vectors: Highly efficient vectors with an enhanced safety profile. Gene Ther..

[111] Leggiero E., Astone D., Cerullo V., Lombardo B., Mazzaccara C., Labruna G., Sacchetti L., Salvatore F., Croyle M., Pastore L. (2013). PEGylated helper-dependent adenoviral vector expressing human Apo A-I for gene therapy in LDLR-deficient mice. Gene Ther..

[112] Wonganan P., Clemens C.C., Brasky K., Pastore L., Croyle M.A. (2010). Species differences in the pharmacology and toxicology of pegylated helper-dependent adenovirus. Mol. Pharm..

[113] White G.I., Monahan P.E. (2007). Gene Therapy for Hemophilia A.

[114] Mitrani E., Pearlman A., Stern B., Miari R., Goltsman H., Kunicher N., Panet A. (2011). Biopump: Autologous skin-derived micro-organ genetically engineered to provide sustained continuous secretion of therapeutic proteins. Dermatol. Ther..

[115] Manno C.S., Pierce G.F., Arruda V.R., Glader B., Ragni M., Rasko J.J., Ozelo M.C., Hoots K., Blatt P., Konkle B. (2006). Successful transduction of liver in hemophilia by AAV-Factor IX and limitations imposed by the host immune response. Nat. Med..

[116] Nathwani A.C., Tuddenham E.G., Rangarajan S., Rosales C., McIntosh J., Linch D.C., Chowdary P., Riddell A., Pie A.J., Harrington C. (2011). Adenovirus-associated virus vector-mediated gene transfer in hemophilia B. N. Engl. J. Med..

